# Survival strategies of planktonic organisms in alpine lakes and beyond: Baldi Memorial Award Lecture presented at the 37th Congress of the International Society of Limnology

**DOI:** 10.1080/20442041.2025.2497248

**Published:** 2025-04-29

**Authors:** Ruben Sommaruga

**Affiliations:** Department of Ecology, Universität Innsbruck, Innsbruck, Austria

**Keywords:** dual phototrophy, global change, photoprotective compounds, polinton-like and giant viruses, proton-pumping rhodopsins

## Abstract

Recent research on alpine lakes has revealed both novel adaptive strategies and established ones, now examined from a new perspective, that planktonic organisms use to persist under the harsh environmental conditions characteristic of these ecosystems. The 2024 Edgardo Baldi lecture addresses 3 major topics, namely, the multifactorial drivers of photoprotection, emerging mechanisms of microbial solar energy utilization, and virus-mediated facilitation of host survival. Photoprotective phenotypes, including the accumulation of mycosporine-like amino acids and carotenoids, are increasingly recognized as outcomes of complex interactions among ultraviolet radiation exposure, nutrient (particularly nitrogen) availability, temperature fluctuations, and predation pressure. Deciphering this interplay is critical for predicting planktonic responses to ongoing global environmental change. A significant advance in the understanding of microbial photoenergetics has been the recent discovery of dual phototrophy in certain bacteria that combine aerobic anoxygenic photosynthesis with rhodopsin-based proton pumping. This dual mechanism enhances energy acquisition and biomass production under conditions of fluctuating light intensity and limited dissolved organic carbon concentrations, with potential parallels in eukaryotic phytoplankton. Finally, the identification of novel viral groups in alpine lakes, such as Polinton-like viruses and virophages, suggests a protective role against host mortality induced by giant viruses. These interactions seem to stabilize microeukaryotic populations and may significantly influence ecosystem productivity and dynamics. Together these findings emphasize the ecological and evolutionary significance of such adaptations, extending beyond alpine lakes to other aquatic ecosystems, and improving our understanding of how plankton respond to environmental changes.

A lake is the landscape’s most beautiful and expressive feature. It is Earth’s eye; looking into which the beholder measures the depth of his own nature.

—Henry David Thoreau

## Research on alpine lakes at the cradle of limnology, yet still poorly known

Scientific interest in lakes located at high elevation dates back to the earliest days of limnology. Foundational studies include Pesta’s (1929) characterization of high mountain lakes in the Austrian Alps, Hutchinson’s (1933) work in ecosystems lying at elevations between 4267 and 5274 m in North India, and Baldi’s (1930) pioneering research on zooplankton in the Monte Rosa region of the Pennine Alps. These early contributions laid the groundwork for the development of alpine limnology as a distinct subfield. However, a significant gap of nearly 60 years occurs before research on these ecosystems began to gain momentum ∼1990. Since then, the number of publications has increased exponentially, although studies on alpine lakes still represent only a small fraction of the overall limnological literature. For example, a search in the Web of Sciences using “alpine, high mountain, high-altitude, or high-elevation AND lakes” results in a maximum of ∼135 publications in 2021. At first sight, this number is surprising given that mountains are often referred to as the “water towers of the world,” providing crucial ecosystem services for a large portion of the global population (Viviroli et al. 2007). Mountains are also known for their high species and genetic diversity and have been key to understanding evolutionary adaptations. However, research conducted in mountains, particularly at high elevation, presents logistical challenges, including access to remote or steep areas and sometimes human acclimatization to low oxygen levels. These factors and associated risks, in addition to low temperatures and long periods of freezing, are certainly a constraint for their study. Further, these conditions also make the proper functioning and maintenance of in-lake sensors for real-time monitoring of water parameters a challenge, although this is also valid in other “extreme” ecosystems. Thus, it is not surprising that our understanding of mountain lakes, particularly at high elevation worldwide, remains highly fragmented. Data gaps and uneven sampling efforts across spatial scales are common and hinder comprehensive assessments of alpine lake ecosystems. Furthermore, experimental work on alpine lakes is particularly scarce, especially outside Europe and North America, where research infrastructure is limited. As a result, important knowledge gaps persist, some of which are also common to lake ecosystems more broadly. Most notably among these is a limited mechanistic understanding of how global stressors such as climate warming, shifting precipitation patterns, acid deposition, and long-range atmospheric transport of pollutants, along with local pressures, affect these ecosystems. While both paleo- and contemporary limnological studies have documented shifts in alpine lake biodiversity linked to these environmental changes, the underlying mechanisms driving these changes often remain unclear.

One promising approach to addressing these knowledge gaps is to study the mechanisms of adaptation in key alpine lake species and functional groups, along with the factors that control their effectiveness. Understanding the mechanisms behind adaptations to harsh environmental conditions such as found in alpine lakes helps explain why species not only thrive, but are competitive enough to form stable populations instead of being excluded by abiotic stress or outcompeted. Adaptations are in a way like fingerprints left during evolution by environmental stressors. In addition, such research can help develop and test predictive models of ecosystem responses to environmental change. Accordingly, the objective of this review is to highlight recent advances in our understanding of adaptation processes within the alpine lake biota. Specifically, I will discuss 3 key topics: first, the multifactorial drivers that operate in plankton to regulate phenotypical traits such as the synthesis and accumulation of photoprotective compounds that mitigate the harmful effects of solar ultraviolet (UV) radiation; second, newly discovered mechanisms by which prokaryotic and potentially eukaryotic microbes harness solar energy to sustain production; and third, the emerging recognition of “protective” viruses as crucial for the survival of phytoplankton and other microeukaryotic hosts. Although none of these mechanisms are exclusive to alpine lakes, and their widespread occurrence in other aquatic ecosystems underscores their evolutionary and ecological significance, these findings are rooted in research conducted in alpine lakes (see more comprehensive coverage of other well-established adaptations of aquatic organisms to high-elevation environments in Jacobsen and Dangles 2017).

## What qualifies as “alpine”?

Before delving into these topics, it is important to clarify a common source of confusion regarding the classification of these lakes in the scientific literature. What exactly qualifies as “alpine”? And why do some publications use terms such as “high mountain” or “high elevation (altitude, which is not the same as elevation, but used as counterpart of latitude) lakes” instead? Part of the different terminology arises from efforts to avoid confusion between lowland lakes located in the Alps (i.e., Alpine with capital A) from true “alpine” lakes. Therefore, the term “high mountain” has been more widely used in Europe, whereas “alpine” is more commonly used in North America and elsewhere. Yet, the definition of alpine comes from terrestrial ecology and refers to the zone in a mountain above the regional treeline, which is defined as the uppermost limit of tree growth (Körner and Hoch 2023, but see Malanson 2024). The treeline represents an important ecotone that changes the strength of connectivity between aquatic ecosystems and their terrestrial surroundings. Above treeline, terrestrial vegetation becomes increasingly sparse particularly in the temperate zone, which in turn affects crucial limnological characteristics such as the typically low concentration of allochthonous chromophoric dissolved organic matter (CDOM) and the resulting high water transparency to solar UV radiation, at least in alpine lakes of the temperate zone (Laurion et al. 2000, Rose et al. 2015, Sommaruga 2001). Yet, because the treeline position varies with latitude and is largely dependent on climatic variables such as air temperature and other local factors that determine tree growth, “alpine” is not strictly tied to a specific elevation (Körner and Hoch 2023). Therefore, defining alpine lakes solely based on the treeline is not universally applicable. For instance, lakes in the high Altiplano of northern Chile, located at elevations ∼4500 m, do not conform this criterion because forests are absent below this region, and therefore the treeline cannot serve as a meaningful ecological boundary. Nevertheless, these lakes share many of the same features typically associated with alpine lakes, such as their glacial origin, cold water temperatures, low nutrient availability, and high water transparency, when not influenced by glaciers. These similarities underscore the limitations of a treeline-based definition and perhaps indicate the need for a different approach to classify alpine-like lakes across diverse geographic contexts. Further, the above arguments make it clear that “high elevation” does not always equate to “alpine,” and setting a universal elevation threshold for defining “high” is problematic; for example, fixed cutoffs such as >500 m (Zhang et al. 2025) can be misleading.

Körner et al. (2011) emphasized that the only universally applicable characteristic of mountains is their steepness, specifically the slope angle relative to the horizontal. In line with this perspective, I propose to refer in future to just mountain lakes and indicate whether they are located above or below the treeline, when present. This approach accommodates regions where forests are naturally absent, such as high tropical plateaus, and avoids the limitations of treeline-based definitions. To maintain consistency, I also suggest adopting the definition of mountainous terrain based on ruggedness, a quantifiable proxy for slope, using thresholds established by the Mountain Biodiversity Initiative (Körner et al. 2011). This approach offers a globally standardized framework for identifying mountain environments based on topographic complexity (Sayre et al. 2018).

## Mitigating the harmful effects of solar UV radiation: from molecules to global change

Solar UV radiation (280–320 nm) is a significant stressor at high elevation sites, where incident levels are markedly elevated, especially relevant for the biota of clear alpine lakes where UV radiation can penetrate deeply into the water column, sometimes reaching even the sediment at biologically meaningful intensities (Sommaruga 2001). This stressor is also particularly important because underwater UV radiation levels are strongly influenced by changes in the duration and optical properties of the ice/snow cover as well as by variations in the concentration of CDOM, both of which are sensitive to climate warming (Sommaruga and Psenner 1997).

In 1994, I discovered the presence of UV-absorbing compounds in plankton collected from the alpine lake Gossenköllesee. At the time, I was assessing temporal changes in UV radiation attenuation during the ice-free season, with a particular focus on the development of the deep chlorophyll *a* maximum, a characteristic feature of many clear aquatic systems (Cullen 1982). Unexpectedly, UV attenuation significantly increased, with a more pronounced peak at ∼340 nm forming within 2 weeks, especially in the deeper water layers where chlorophyll *a* also increased (Sommaruga and Psenner 1997). This increase could not be accounted for by changes in dissolved organic carbon concentrations or by the typical absorption pattern of CDOM (Sommaruga and Psenner 1997), suggesting that UV absorbing compounds within the plankton were strongly absorbing these specific wavelengths. However, identifying these unknown compounds took time, until we eventually discovered they were mycosporine-like amino acids (MAAs; Sommaruga and Garcia-Pichel 1999), a discovery that represents the first identification of MAAs in freshwater organisms, although they were known from marine organisms.

**Figure 1.**
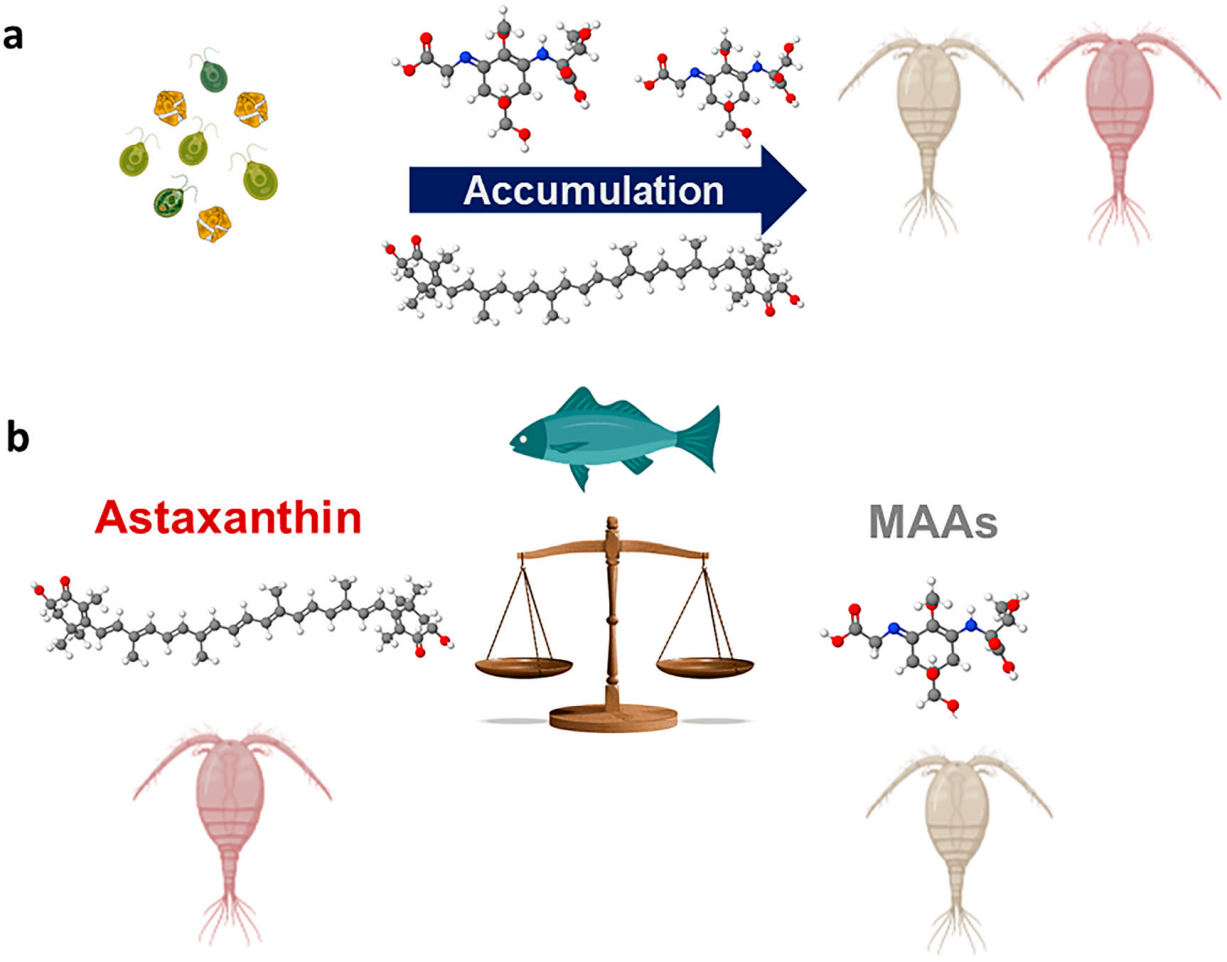
Accumulation and balance of photoprotective compounds in zooplankton. (a) Phytoplankton produce mycosporine-like amino acids (MAAs) and carotenoids, which subsequently accumulate in copepods through trophic transfer. (b) Copepods regulate the balance between MAAs (gray) and astaxanthin (red) under the influence of visually oriented planktivore fish. The scale symbolizes the trade-off or equilibrium between these 2 compounds. Color-coded dots in molecules represent nitrogen (blue), carbon (grey), oxygen (red), and hydrogen (white). Molecules were designed with Jmol, and illustrations are from Biorender.

MAAs are water soluble secondary metabolites with strong UV absorption efficiency because of their high molar extinction coefficient and are often referred to as “invisible sunscreens” because they are not pigments. Although primarily produced by algae and other microbes, they can accumulate in organisms unable to synthesize them, such as copepods (Fig. 1a), through their diet (Moeller et al. 2005, Hylander and Jephson 2010). One of the initial questions that emerged was how much variability exists in MAA accumulation among copepod populations, which can retain these compounds for significantly longer periods (weeks) than phytoplankton. Field studies on the copepod *Cyclops abyssorum tatricus* have shown a strong relationship between MAA concentrations and lake UV transparency (Tartarotti et al. 2001). Notably, the depth refuge, defined as the ratio of UV penetration (e.g., at 320 nm) to the lake’s maximum depth, explained 86% of the variation in MAA concentrations across lakes at different elevations, emphasizing UV exposure as a key driver of MAA accumulation (Sommaruga 2001, 2010).

Laboratory experiments further demonstrated the superior photoprotective efficacy of MAAs compared with other photoprotective compounds such as carotenoids, which are also accumulated by copepods (Fig. 1a). In experiments using 2 phenotypes of the copepod *Leptodiaptomus minutus—*one containing carotenoids but lacking MAAs and the other with MAAs but no carotenoids—only the MAA-containing individuals survived and even exhibited growth under UV exposure. By contrast, copepods lacking MAAs experienced significant mortality (Moeller et al. 2005).

Secondary carotenoids such as astaxanthin (sometimes complexed with esters or proteins) are also key photoprotective compounds in copepods, although their function differs from that of MAAs. Carotenoids are pigments, and many act as strong antioxidants, mitigating oxidative and photooxidative stress. However, unlike MAAs, carotenoids do not efficiently block UV wavelengths, and at high concentrations their red/orange/blue hue can pose a risk to copepods by increasing their visibility to visual predators (Hansson et al. 2007). The presence of visually foraging predators such as fish seems to influence the balance (Fig. 1b) between carotenoids and MAA accumulation in copepods (Hylander and Hansson 2013, Oester et al. 2022). For instance, the community composition of predatory fish determines the levels of astaxanthin in individuals of *L. minutus* (Oester et al. 2022), although the underlying mechanism for this trade-off remains unresolved. In fishless lakes, such as those found at high elevations in the Himalayas, copepods exhibit the highest recorded carotenoid concentrations (Sommaruga 2010). Interestingly, despite the extreme UV conditions at elevations >4500 m in the Himalayas, MAA concentrations were undetectable in phytoplankton and found at low concentrations in copepods (Sommaruga 2010). This discrepancy led to the hypothesis that additional factors, particularly nutrient availability, may also be important in regulating MAA synthesis and the subsequent accumulation by zooplankton.

One approach to exploring this hypothesis was to examine the potential for nutrient limitation, with a focus on nitrogen. Nitrogen (N) availability is likely to influence the balance between MAA and carotenoids production because MAAs are nitrogenous compounds, whereas carotenoids such as astaxanthin are N-free and composed primarily of carbon. Studies on marine primary producers have demonstrated that N availability influences MAA synthesis rates (Peinado et al. 2004, Korbee et al. 2010), evidence that aligns with that of high elevation tropical lakes where N is usually limiting (Vincent et al. 1984) and no detectable MAAs in phytoplankton and zooplankton are found (Kinzie et al. 1998). Moreover, studies have shown that N limitation, combined with high light exposure, promotes the straightforward synthesis of secondary carotenoids such as astaxanthin in algal cultures (Wang et al. 2021, Li et al. 2022).

**Figure 2.**
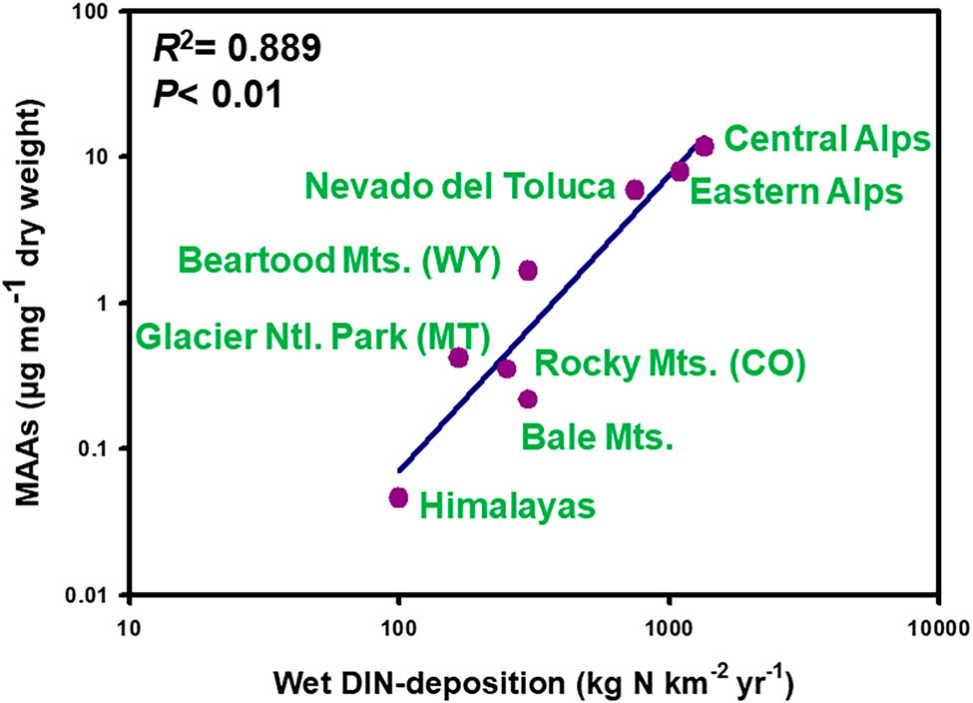
Relationship between mycosporine-like amino acids (MAAs) concentrations in copepod populations from high elevation lakes in different mountain regions and average wet dissolved inorganic nitrogen (DIN) atmospheric deposition rates.

To test the idea of the effect of N availability on MAAs accumulation in zooplankton, I used annual wet inorganic N-deposition rates (Dentener et al. 2006) as a proxy and correlated them with available MAA concentration data for different copepod populations of alpine lakes (data sources in Supplemental Material). Despite differences in copepod species, the limited number of lakes, and the coarse approximation of N-deposition rates, the correlation was strikingly strong (*R*² = 0.889, *p* < 0.01), with the highest MAA levels found in N-rich regions like the Alps and the lowest found in N-poor regions like the Himalayas (Fig. 2). Although experimental validation with natural phytoplankton communities in N-limited lakes is still needed to establish a proof of concept, it is plausible that atmospheric N pollution has influenced the past, present, and future capacity of phytoplankton to efficiently synthesize MAAs. While atmospheric N pollution is declining in parts of Europe, it remains a significant concern in many regions worldwide, with potential far-reaching implications for alpine lake ecosystems (Wolfe et al. 2003, Holtgrieve et al. 2011, Hundey et al. 2016, Moser et al. 2019).

**Figure 3.**
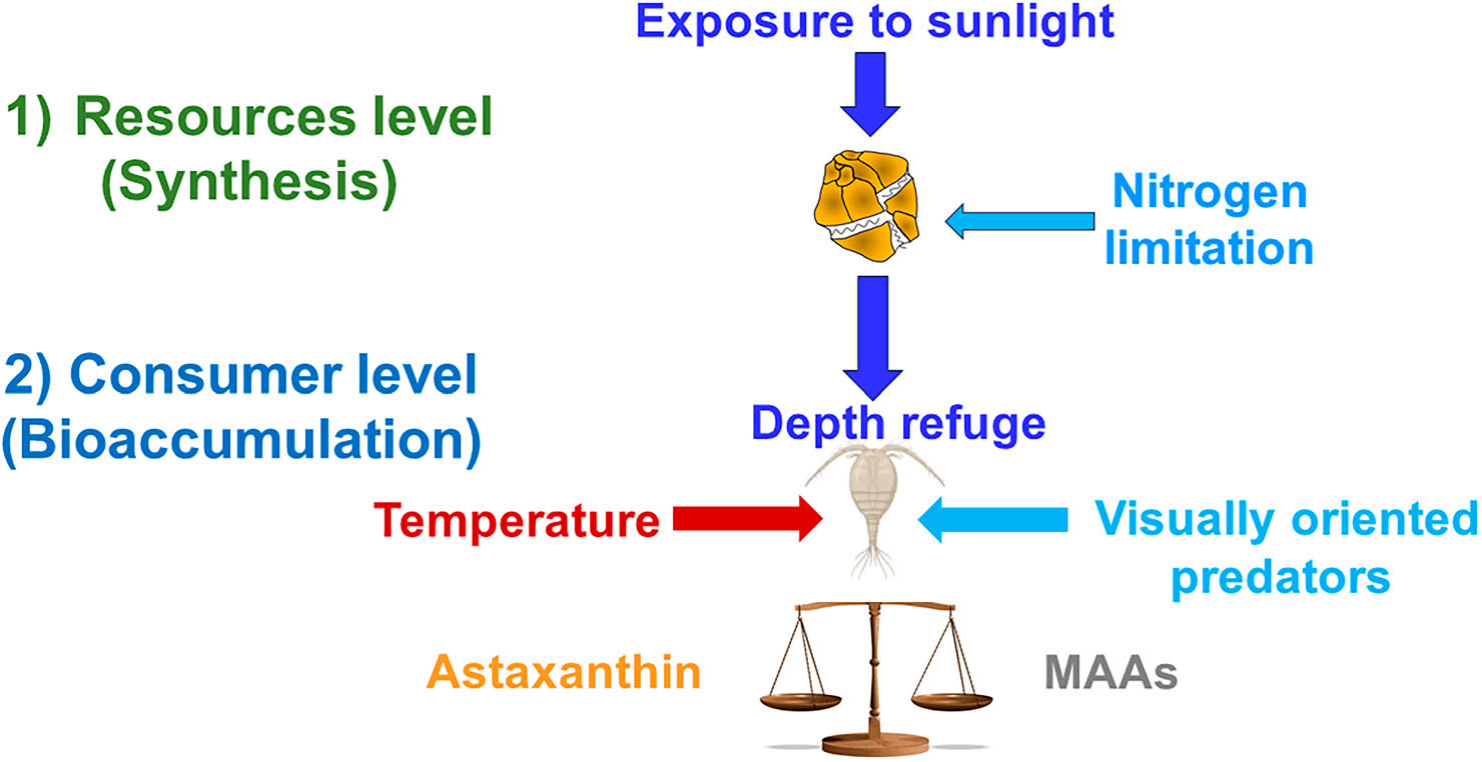
The interplay between resource-level synthesis and consumer-level regulation of photoprotective compounds in planktonic organisms is shaped by selective pressures and trade-offs. At the resource level, sunlight exposure (dark blue arrow) drives the production of photoprotective compounds in primary producers (e.g., phytoplankton). However, nitrogen limitation (light blue arrow) influences the relative synthesis of astaxanthin and mycosporine-like amino acids (MAAs). These resource-level dynamics, in turn, impact the balance of these compounds at the consumer level. In addition, bioaccumulation levels of MAAs by copepods are affected by the lake depth refuge (dark blue arrow), temperature variations (red arrow), while the presence of visually oriented predators (light blue arrow) affects astaxanthin levels.

These findings indicate a 2-tiered control mechanism regulating MAA accumulation in copepods. First, sunlight exposure and N availability drive MAA synthesis in phytoplankton while N limitation shifts the balance toward astaxanthin production, resulting in its subsequent dietary accumulation by copepods (Fig. 3). Second, ecological and environmental factors such as predation (Hylander et al. 2009, Oester et al. 2022), UV depth refuge (Sommaruga 2010), and temperature (Garcia et al. 2008) further modulate MAA accumulation levels in copepods. This interplay exemplifies the complex adaptations and trade-offs that shape survival strategies in alpine lakes.

## Dual phototrophy: the “hybrid motor” of microbes to efficiently use solar energy

**Figure 4.**
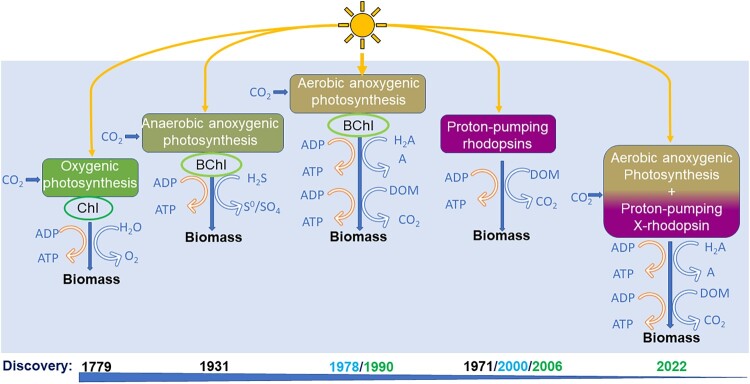
Evolution of diverse mechanisms for harvesting solar energy in photo(hetero)trophic organisms. The timeline at the bottom highlights key discoveries of these processes and underscores the progressive understanding of solar energy utilization in diverse ecosystems, from oxygenic photosynthesis (1779) to dual phototrophy (2022). Years in dark blue indicate the original description of the mechanism, in turquoise denote the first discovery in marine ecosystems, and green represent the first discovery in freshwater ecosystems. Modified from Karl (2002). ADP: adenosine diphosphate, ATP: adenosin triphosphate, Bchl: bacteriochlorophyll, Chl: chlorophyll, DOM: dissolved organic matter, H_2_A: acceptor other than water, X-rhodopsin: xanthorhodopsin.

Our knowledge about the various mechanisms of phototrophic organisms to harvest solar energy and produce biomass has changed considerably in recent decades and continues to evolve (Fig. 4). For a long time, oxygenic photosynthesis was recognized as the only mechanism to create new biomass in primary producers (Ingen-Housz 1779, but Hill 1937 first reported the mechanism). However, another mechanism, anoxygenic photosynthesis, was detected later (Van Niel 1932), although it certainly appeared earlier in the evolution of photosynthesis (Bendall et al. 2008). This mechanism does not produce oxygen because it uses other electron donors, such as H_2_S, instead of water. Initially, anoxygenic photosynthesis was thought to occur only under anaerobic conditions. However, starting with the finding in marine (Harashima et al. 1978, Kolber et al. 2001) and later freshwater (Yurkov and Gorlenko 1990, Eiler 2006) ecosystems, microorganisms were found containing bacteriochlorophyll *a* capable of anoxygenic photosynthesis in aerobic aquatic environments (Fig. 4). These microorganisms are mixotrophic, meaning in this case that they can use light to generate energy while simultaneously assimilating dissolved organic carbon for growth (Fig. 4). The fourth mechanism, discovered by Oesterhelt and Stoeckenius (1971) involves proton-pumping rhodopsins (PPRs), simple proteins that use light energy to pump protons across membranes, creating a gradient that drives ATP synthesis and sustains growth. Much later, aquatic microorganisms were found to use PPRs to generate energy from solar radiation in marine (Béjà et al. 2000) and freshwater (Sharma et al. 2006) ecosystems (Fig. 4).

Both aerobic anoxygenic photosynthesis and rhodopsin-based phototrophy genes have been identified in the genomes of numerous microorganisms (Kopejtka et al. 2020, Zeng 2023). However, the proof of concept that bacteria are capable of using more than one mechanism to harvest solar energy and produce biomass, a phenomenon called “dual phototrophy,” was only recently demonstrated (Kopejtka et al. 2022).

In a study conducted in alpine and subalpine lakes, aerobic anoxygenic phototrophic bacteria comprised a significant portion of the total bacterial biomass (Čuperová et al. 2013). Among these bacteria, *Sphingomonas glacialis* was isolated and used to demonstrate the advantages and existence of dual phototrophy (Kopejtka et al. 2021, 2022). Experiments with this isolate demonstrated, for example, that in the strain containing the PPR xanthorhodopsin but lacking bacteriochlorophyll *a*, respiration rates decreased significantly with increasing light (Kopejtka et al. 2022). Additionally, light exposure led to more efficient ATP production compared to dark controls (Kopejtka et al. 2022). Growth yield under light conditions also surpassed that in darkness, with bacterial abundance reaching higher levels by the experiment’s end. These findings demonstrated that PPR in this isolate is highly efficient and crucial for bacterial metabolism, particularly in oligotrophic ecosystems such as alpine lakes, where dissolved organic carbon is scarce.

A key distinction between PPRs and photosynthetic pigments like chlorophyll *a* or bacteriochlorophyll *a* lies in their absorption of different parts of the solar spectrum. Rhodopsins primarily absorb light at wavelengths ∼570 nm, with minimal overlap with other pigments, suggesting that their evolutionary incorporation enhanced energy utilization efficiency by reducing competition for light (Sephus et al. 2022). However, the absorption properties of rhodopsins can be modulated by the presence of carotenoid antennae (Imasheva et al. 2009, Chazan et al. 2023), as observed also in *S. glacialis* (Kopejtka et al. 2022).

How dual phototrophy operates under natural conditions remains an active area of investigation, but it is likely driven by distinct seasonal factors influencing the synthesis of the 2 phototrophic systems (Kopejtka et al. 2022). According to the authors’ hypothesis, during the short winter days when light penetration into the water column is significantly reduced by snow and ice cover, the synthesis of bacteriochlorophyll *a* is favored because of its adaptation to low-light conditions. By contrast, from late spring to early summer, as ice melts and light availability increases, PPRs likely become advantageous because of their simpler biosynthesis and efficient function under higher light intensities. During summer, both phototrophic systems are presumed to operate concurrently. As autumn approaches, decreasing photoperiods and cooler temperatures may again promote bacteriochlorophyll *a* synthesis. This seasonal pattern is consistent with field observations made in one alpine lake, where the abundance of aerobic anoxygenic phototrophic bacteria peaks in autumn (Čuperová et al. 2013).

The prevalence of dual phototrophy across natural microbial communities remains an open question, but several microbial genomes have been identified containing genes for both phototrophic mechanisms (Zeng 2023). Demonstrating the concurrent activity of both mechanisms in these microbes is challenging, but while dual phototrophy has been experimentally confirmed only in a bacterium isolated from an alpine lake, it is likely a more general phenomenon not restricted to this environment. Microorganisms possessing genes for both phototrophic pathways have been detected in diverse ecosystems, including desert lakes and hot springs, suggesting a broader distribution (Zeng 2023).

**Figure 5.**
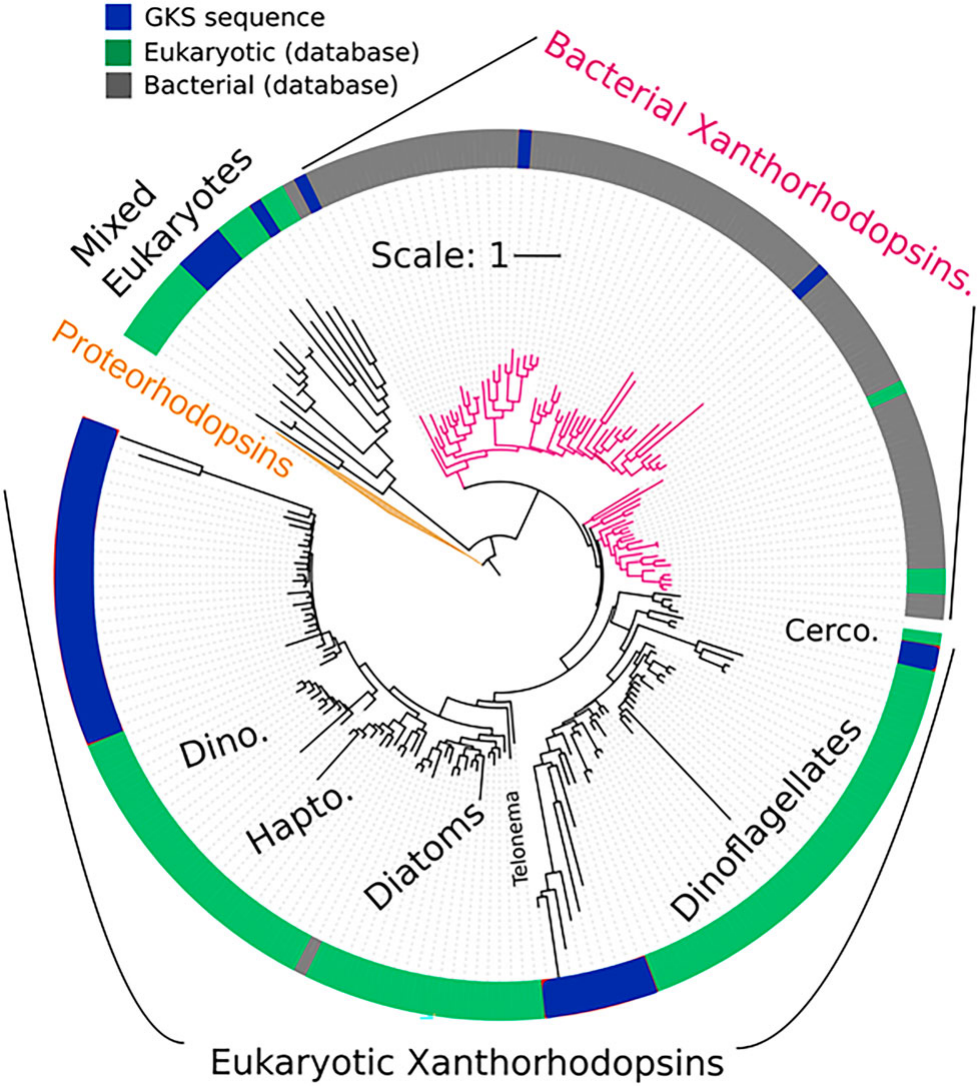
Phylogenetic tree of xanthorhodopsins and related rhodopsins in Gossenköllesee. Colored branches and labels highlight key groups: bacterial xanthorhodopsins (pink), proteorhodopsins (orange, collapsed group), and mixed eukaryotic sequences (black). The outer ring categorizes sequences into 3 groups: GKS sequences (blue), eukaryotic database sequences (green), and bacterial database sequences (gray). Major taxonomic groups, including dinoflagellates (Dino.), diatoms, haptophytes (Hapto.), and cercozoans (Cerco.), are labeled around the tree. The scale bar represents one amino acid substitution on average per site.

The discovery of dual phototrophy in bacteria also raised the question of whether eukaryotic microbes such as phytoplankton might also integrate oxygenic photosynthesis with PPRs. Genomic analyses of phytoplankton (details in Supplemental Material) from the alpine lake Gossenkölle have revealed the presence of genes for eukaryotic PPRs in dinoflagellates, cryptophytes, and diatoms (Fig. 5). While the mere presence of these genes does not confirm functional activity, it provides plenty of opportunities for future research. In fact, recent studies in marine ecosystems suggest that PPRs could be a crucial and common energy-producing strategy in phytoplankton. For example, Strauss et al. (2023) demonstrated the widespread distribution of PPR genes among marine phytoplankton while Andrew et al. (2023) showed that PPRs alone could sustain phytoplankton biomass production in polar waters. Furthermore, Zepernick et al. (2024) recently reported that freshwater diatoms in Lake Erie expressed genes for PPRs under turbid summer conditions, further supporting the potential ecological significance of PPRs in diverse environments.

Unlike chlorophyll *a*–based systems, PPRs seem to function efficiently not only when light inhibits photosystem II (Andrew et al. 2023), but also under low-light conditions where photosynthesis is not saturated (Zepernick et al. 2024). Additional factors such as low temperature and nutrient limitation, particularly by phosphorus, may further favor a shift toward energy production via PPRs (Ma et al. 2024). This emerging evidence questions traditional views on primary production in aquatic ecosystems, although a proof of concept for freshwater phytoplankton is still needed. Current methods to measure primary production, such as oxygen production or inorganic carbon uptake, are not applicable to rhodopsin-based phototrophy. Consequently, the development of new methods will be crucial for quantifying the contribution of this mechanism to biomass production. In the coming years, significant research efforts are expected to focus on unraveling the ecological importance of dual phototrophy across diverse aquatic ecosystems and taxa. This line of investigation could revolutionize our understanding of how solar energy, beyond classical oxygenic photosynthesis, is harnessed in aquatic environments.

Building on this, a compelling transition to the next chapter involves the role of giant viruses, which may have facilitated the spread of genes encoding proteorhodopsins (PPRs) among marine and freshwater microeukaryotes (Needham et al. 2019, Hososhima et al. 2022). These viruses represent an intriguing avenue of study, potentially linking viral ecology with novel phototrophic capabilities in aquatic systems.

## Viruses: the enemy of my enemy is my friend

Viruses are not only the most abundant biological entities across virtually all environments, but they also serve as key agents of mortality for nearly all aquatic organisms. Through cell lysis, viruses redistribute biochemical compounds from the host, influencing global biogeochemical cycles of carbon and nutrients (Suttle 2005). Furthermore, viruses shape planktonic communities by regulating the abundance and diversity of different populations while also modulating host metabolism via viral-encoded auxiliary metabolic genes (Luo et al. 2022). While viruses are widely recognized as a significant cause of mortality in aquatic ecology, not all viruses cause immediate death (e.g., those with a lysogenic life cycle), and ongoing research continues to uncover new viral life cycles and groups (Correa et al. 2021). Despite their ecological significance, viruses have been less studied in freshwater than marine environments.

Our research group began investigating viruses in alpine lakes 24 years ago. During this time, we discovered large filamentous virus-like particles in several alpine lakes suspected to be large bacteriophages (Pina et al. 1998, Hofer and Sommaruga 2001). To this day, these filamentous viruses remain uncharacterized and do not fit into any known virus group. At the time of their detection, molecular tools to characterize viruses were limited, but in the era of metagenomic analyses, we now know that alpine lakes harbor diverse virus communities, including giant viruses (phylum Nucleocytoviricota) that infect microeukaryotes. These viruses are, as their name implies, exceptionally large and usually exceed 400 nm, making them several times larger than bacteriophages found in aquatic ecosystems (Schulz et al. 2022). Alpine lakes are ideal for the detection of novel viruses because their low diversity, compared to other more productive systems, facilitates the assembly of longer contigs, and even complete virus genomes. For example, abundant Polinton-like viruses have recently been identified in alpine lakes, thought to be associated with and dependent on giant viruses for their replication (Bellas and Sommaruga 2021). Recent research on alpine lakes has identified the genomes of tens of thousands of novel Polinton-like viruses and virophages found to be widespread among microeukaryotes, including phytoplankton, that are often integrated into the genomes of their hosts (Bellas et al. 2023). Interestingly, these viruses are not confined to freshwater ecosystems but are also found in marine environments, emphasizing their broad ecological significance (Bellas and Sommaruga 2021).

One of the most ecologically important functions of most Polinton-like viruses is their likely role in preventing or minimizing host mortality caused by giant viruses in microeukaryotes, a phenomenon recently demonstrated for the first time in a phytoplankton species (Roitman et al. 2023). This “protective” role is similar to that of virophages (family Lavidaviridae), small viruses that target giant viruses, thus protecting their microeukaryotic hosts (Fischer and Hackl 2016, Duponchel and Fischer 2019), but Polinton-like viruses seem to be more prevalent in aquatic microeukaryotes than virophages (Bellas et al. 2023). Both Polinton-like viruses and virophages can independently infect a host, integrate into its genome, and later reactivate to parasitize on giant viruses (Moniruzzaman and Aylward 2023, Roitman et al. 2023). Alternatively, they can co-infect the host alongside the giant virus to achieve the same effect (Moniruzzaman and Aylward 2023). Although our comprehension of interactions between hosts and those viruses in their natural habitat is still developing, emerging techniques such as single-cell RNA sequencing are promising to allow the analysis of both viral-host interactions and their temporal dynamics (Fromm et al. 2024).

The interactions between a microeukaryotic host and 2 different types of viruses exemplify what is likely one of the oldest forms of tripartite coevolution (Barreat and Katzourakis 2023), with probably significant ecological implications. For instance, the presence of virophages is known to dramatically alter phytoplankton population dynamics (Yau et al. 2011). In their numerical model, Yau et al. (2011) found that when only an algal population and a giant virus interact, the algal population is severely decimated, but when virophages are present the algal population becomes more stable and the system’s turnover increases. This process, in turn, likely has positive effects on bacterial secondary production through the release of dissolved organic matter from algae killed by the virus. In oligotrophic ecosystems, where host abundance is low, strategies like this may have evolved to tightly regulate mortality rates, ensuring that populations are not completely eliminated. This control mechanism likely reflects evolutionary pressures in nutrient-poor environments, although the consequences for ecosystem functioning remain to be studied.

## Conclusions

Alpine lakes, despite the logistical challenges they present, serve as valuable natural models for understanding the adaptations of planktonic organisms to harsh environmental conditions and their responses to various global change drivers. The findings presented here demonstrate the complexity of these adaptations, their underlying drivers, and their broader ecological significance for other aquatic ecosystems. One example is the intricate interplay of factors regulating the balance of photoprotective compounds synthesized by phytoplankton and subsequently accumulated by copepods, where N availability seems to be crucial. The discovery of dual phototrophy in bacteria represents a significant advancement in our understanding of microbial strategies for efficiently harnessing solar energy, while recent evidence suggesting its potential occurrence in eukaryotic phytoplankton further highlights the gaps in our knowledge of primary production and energy acquisition. Lastly, the role of “protective” viruses adds another dimension to ecosystem complexity, shaping microeukaryote population dynamics and their interactions with giant viruses.

In light of the accelerating impacts of climate change and other global stressors, uncovering the adaptive mechanisms of lake biota will be crucial for safeguarding the ecological integrity of these vulnerable environments.

## Supplementary Material

Supplemental Material
