# Supplementary material for: Survival strategies of planktonic organisms in alpine lakes and beyond: Baldi Memorial Award Lecture presented at the 37th Congress of the International Society of Limnology
Source: Inland Waters. 2025 Apr 29;15(1):2497248. doi: 10.1080/20442041.2025.2497248 (PMC12306673; doi:10.1080/20442041.2025.2497248)
Supplement: Supplemental Material [file TINW_A_2497248_SM7463.docx]

**Supplementary information**

**Survival strategies of planktonic organisms in alpine lakes and beyond**

Ruben Sommaruga

Universität Innsbruck, Department of Ecology, Technikerstr. 25, 6020 Innsbruck, Austria. [Ruben.sommaruga@uibk.ac.at](mailto:Ruben.sommaruga@uibk.ac.at)

**Data source on N-atmospheric deposition**

Since most of the available data on MAA concentrations in copepods dates back over two decades, I used data on wet nitrogen atmospheric deposition reported by Dentener et al. (2006). Although these values are derived from a coarse model, atmospheric nitrogen deposition rates have undergone significant changes in recent decades. Values from this model were consistent with those reported by Bergström and Jansson (2006) for Europe and North America.

Bergström AK, Jansson M. 2006. Atmospheric nitrogen deposition has caused nitrogen enrichment and eutrophication of lakes in the northern hemisphere. Global Change Biol. 12:635–643. <https://doi.org/10.1111/j.1365-2486.2006.01129.x>

Dentener F, Drevet J, Lamarque JF, Bey I, Eickhout B, Fiore AM, Hauglustaine D, Horowitz LW, Krol M, Kulshrestha UC, Lawrence M, Galy-Lacaux C, Rast S, Shindell D, Stevenson D, Van Noije T, Atherton C, Bell N, Bergman D, Butler T, Cofala J, Collins B, Doherty R, Ellingsen K, Galloway J, Gauss M, Montanaro V, Müller JF, Pitari G, Rodriguez J, Sanderson M, Solmon F, Strahan S, Schultz M, Sudo K, Szopa S, Wild O. 2006. Nitrogen and sulfur deposition on regional and global scales: A multimodel evaluation. Global Biogeochemical Cycles 20: GB2026. <https://doi.org/10.1029/2005GB002672>

**Data sources on MAA concentrations**

Alcocer J, Delgado CN, Sommaruga R. 2020. Photoprotective compounds in zooplankton of two adjacent tropical high mountain lakes with contrasting underwater light climate and fish occurrence. J Plankton Res. 42:105–118. <https://doi.org/10.1093/plankt/fbaa001>

Hylander S. 2020. Mycosporine-Like Amino Acids (MAAs) in Zooplankton. Mar Drugs. 18, 72. <https://doi.org/10.3390/md18010028>

Persaud AD, Moeller RE, Williamson CE, Burns CW. 2007. Photoprotective compounds in weakly and strongly pigmented copepods and co-occurring cladocerans*.* Freshw Biol. 52:1406-1417. <https://doi.org/10.1111/j.1365-2427.2007.01833.x>

Sommaruga R. 2010. Preferential accumulation of carotenoids rather than of mycosporine-like amino acids in copepods from high-altitude Himalayan lakes. Hydrobiologia. 648:143–156. https://doi.org/10.1007/s10750-010-0141-y

Tartarotti B, Laurion I, Sommaruga R. 2001. Large variability in the concentration of mycosporine-like amino acids among zooplankton from lakes located across an altitude gradient. Limnol Oceanogr. 46:1546–1552.

Data on MAAs for the copepods in Lake Garba Guracha, Bale Mts. are from the author (unpublished).

**
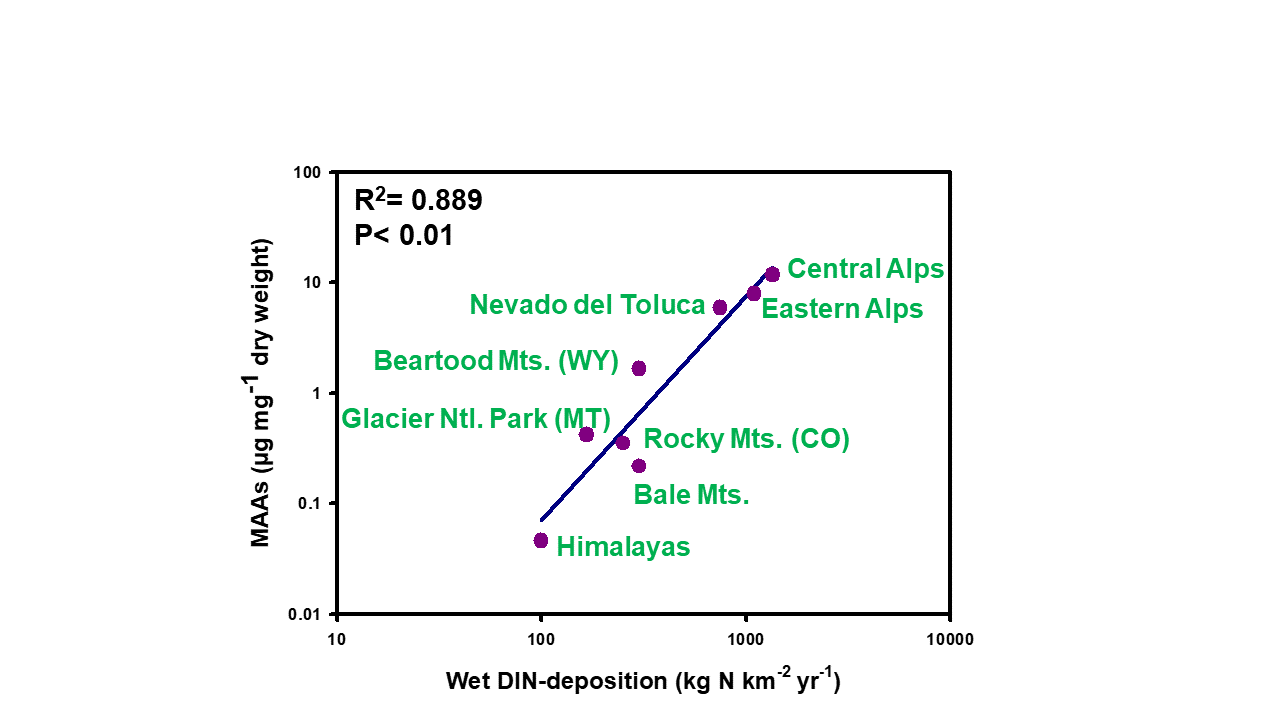
**

**Fig. S1.** Relationship between mycosporine-like aminoacids (MAAs) concentrations in copepod populations from high elevation lakes in different mountain regions and wet dissolved inorganic nitrogen (DIN) atmospheric deposition.

**Method for rhodopsin detection**

For the detection of eukaryotic rhodopsin genes in Gossenköllesee, long metagenomic reads were generated from water samples collected on 5^th^ September 2022. Two litres of water (depth composite sample) was pre-filtered through a 40 µm nylon mesh, before collection on a 47 mm, 3-µm pore size polycarbonate membrane. DNA was extracted from the filter using a Qiagen DNEasy Powerwater kit, before library preparation and sequencing on an Oxford Nanopore Minion Mk1b (R10.4.1 flow cell). Reads were base called via Guppy using the Super Accuracy (SUP) mode to achieve an estimated accuracy of 98-99%. To detect rhodopsin genes, reads were searched (diamond blastx) against a custom database of all published eukaryotic and bacterial rhodopsin genes from GenBank and UniprotKB (evalue cut-off 1e-12, 150aa length). Frameshifts in the reads were corrected using proovframe (Hackl et al, 2021) and matching regions were retrieved as amino acid sequences. Matching rhodopsin reads were aligned using MAFFT E-ins-i (Katoh and Standlet, 2013) along with selected reference rhodopsins from the databases, coupled with those from Strauss et al 2023. A phylogenetic tree was constructed with PhyML using the Geneious Prime software suite.

**References**

Hackl T, Trigodet F, Eren AM, Biller SJ, Eppley JM, Luo E, Burger A, DeLong EF, Fischer MG. 2021. Proovframe: Frameshift-correction for long-read (meta)genomics [Preprint]. bioRxiv. https://doi.org/10.1101/2021.08.23.457338

Katoh K, Standley DM. 2013. MAFFT Multiple Sequence Alignment Software Version 7: Improvements in Performance and Usability. Mol Biol Evol. 30:772–780. <https://doi.org/10.1093/molbev/mst010>

Strauss J, Deng L, Gao S. et al. 2023. Plastid-localized xanthorhodopsin increases diatom biomass and ecosystem productivity in iron-limited surface oceans. Nat Microbiol. 8:2050–2066. <https://doi.org/10.1038/s41564-023-01498-5>
